# Evaluating the Impact of Spinach Supplementation in Enhancing the Growth of Blue Swimming Crab Larvae, *Portunus pelagicus*

**DOI:** 10.21315/tlsr2026.37.1.4

**Published:** 2026-03-31

**Authors:** Alias Redzuari, Ariffin Hidir, Muyassar H. Abualreesh, Hanafiah Fazhan, Khor Waiho, Hongyu Ma, Mhd Ikhwanuddin

**Affiliations:** 1 Higher Institution Centre of Excellence (HICoE), Institute of Tropical Aquaculture and Fisheries, Universiti Malaysia Terengganu, 21030 Kuala Nerus, Terengganu, Malaysia; 2 Department of Marine Biology, Faculty of Marine Sciences, King Abdulaziz University, Jeddah 21589, Saudi Arabia; 3 STU-UMT Joint Shellfish Research Laboratory, Shantou University, Shantou, China; 4 Guangdong Provincial Key Laboratory of Marine Biotechnology, Shantou University, Shantou, China

**Keywords:** *Portunus pelagicus*, *Spinacia oleracea*, Crab Larvae, Growth, Steroid, Portunus pelagicus, Spinacia oleracea, Larva Ketam, Pertumbuhan, Steroid

## Abstract

Blue swimming crabs (*Portunus pelagicus*) consistently command higher prices in domestic and global markets due to elevated demand. Due to the rising issue of low crab larvae survival, farmers have begun using artificial hormones to increase survival rates and ensure hatchery production aligns with market demands. However, the outcomes of using artificial hormones are concerning, as these compounds pose serious risks to aquatic organisms, humans, and the environment. To address the harmful effects of artificial hormones, efforts are now focused on identifying affordable and eco-friendly plant-based alternatives, such as spinach (*Spinacia oleracea*), which contains bioactive compounds that could promote crab larval growth and survival. This study aimed to investigate the efficacy of spinach in improving the growth performance of *P.pelagicus* larvae. This was accomplished by enriching *Artemia* with spinach, allowingit to serve as a nutrient-enhanced live feed. Compounds in the spinach leaf wereextracted with methanol and analysed through GC-MS to identify potential growth-related compounds. LC_50_ analysis (1,000 ppm–2,000 ppm) was conducted on *Artemia* todetermine the optimal enrichment concentration (1,600 ppm) before use in larval feeding.Results indicated spinach contains compounds such as steroids (5.9%), palmitic acid(5.2%), oleic acid (2.5%) and flavonoids (3.6%) that can potentially improve larval growth.Toxicity analysis showed that the 1,600 ppm treatment was the LC_50_, meaning it is theoptimal concentration for *Artemia* enrichment, delivering the highest amount of bioactivecompounds without affecting *Artemia* survival. Larvae were fed spinach-enriched *Artemia*(1,600 ppm) and compared with unenriched *Artemia* (control). The 1,600 ppm treatmentresulted in significantly higher survival at various larval stages (Z1–Z2: 82.6%, Z2–Z3:91.9%, M–C1: 38.1%), shorter intermoult periods for Z2–Z3 (2.9 days), Z3–Z4 (3.1 days),and M–C1 (3.8 days) and higher SGR (M: 22.5% day^−1^, C1: 24.5% day^−1^). Larval steroidanalysis supported these findings, with treatment 1,600 ppm yielding higher steroid levels across all stages (511.63–3953 pg/mL), suggesting that spinach-derived steroids may stimulate moulting and enhance larval growth and survival.

## Abstrak

Ketam renang biru (Portunus pelagicus) mendapat harga yang lebih tinggi di pasaran domestik dan global secara konsisten kerana permintaan yang tinggi. Disebabkan isu peningkatan kadar kematian larva ketam, para penternak telah mula menggunakan hormon tiruan untuk meningkatkan kadar kelangsungan hidup dan memastikan pengeluaran di ternakan sejajar dengan permintaan pasaran. Namun, hasil penggunaan hormon tiruan membimbangkan kerana sebatian ini menimbulkan risiko serius kepada organisma akuatik, manusia dan alam sekitar. Untuk menangani kesan berbahaya hormon tiruan, usaha kini tertumpu kepada mengenal pasti alternatif berasaskan tumbuhan yang mampu milik dan mesra alam, seperti bayam (Spinacia oleracea), yang mengandungi sebatian bioaktif yang boleh merangsang pertumbuhan dan kelangsungan larva ketam. Kajian ini bertujuan untuk mengkaji keberkesanan bayam dalam meningkatkan prestasi pertumbuhan larva P. pelagicus. Ini dicapai dengan memperkayakan Artemia dengan bayam, membolehkan ia berfungsi sebagai makanan hidup yang diperkaya dengan nutrien. Kompaun dalam daun bayam diekstrak menggunakan metanol dan dianalisis melalui GC-MS untuk mengenal pasti kompaun yang berpotensi berkaitan dengan pertumbuhan. Analisis LC_50_ (1,000 ppm–2,000 ppm) dijalankan pada Artemia untuk menentukan kepekatan pengayaan optimum (1,600 ppm) sebelum digunakan dalam pemakanan larva. Hasil menunjukkan bayam mengandungi kompaun seperti steroid (5.9%), asid palmitik (5.2%), asid oleik (2.5%) dan flavonoid (3.6%) yang berpotensi meningkatkan pertumbuhan larva. Analisis ketoksikan menunjukkan rawatan 1,600 ppm adalah LC_50_, bermakna ia adalah kepekatan optimum untuk pengayaan Artemia, memberikan jumlah kompaun bioaktif tertinggi tanpa menjejaskan kelangsungan hidup Artemia. Larva diberi makan Artemia yang diperkaya dengan bayam (1,600 ppm) dan dibandingkan dengan Artemia yang tidak diperkaya (kawalan). Rawatan 1,600 ppm menghasilkan peratusan kelangsungan hidup yang lebih tinggi secara signifikan pada pelbagai peringkat larva (Z1–Z2: 82.6%, Z2–Z3: 91.9%, M–C1: 38.1%), tempoh moult antara yang lebih singkat untuk Z2–Z3 (2.9 hari), Z3–Z4 (3.1 hari), dan M–C1 (3.8 hari) dan Kadar Pertumbuhan Spesifik (SGR) yang lebih tinggi (M: 22.5% sehari, C1: 24.5% sehari). Analisis steroid larva menyokong penemuan ini, dengan rawatan 1,600 ppm menghasilkan tahap steroid yang lebih tinggi di semua peringkat (511.63–3953 pg/mL), mencadangkan bahawa steroid yang diperoleh daripada bayam mungkin merangsang pertukaran kulit dan meningkatkan pertumbuhan serta kelangsungan hidup larva.

**HIGHLIGHTS**

- Spinach contains essential bioactive compounds such as steroids, palmitic acid, oleic acid and flavonoid derivatives.
- The 1,600 ppm treatment of spinach resulted in higher survival at various larval stages.
- The 1,600 ppm treatment resulted in higher steroid levels in crab larvae, stimulating larval moulting.

## INTRODUCTION

Blue swimming crabs, *Portunus pelagicus* found in the Pacific Ocean and valued for their delicacy and larger size, consistently receive higher prices in both domestic and global markets due to their higher demand (Sugumar *et al*. 2013). Crab farming holds significant importance for the economy, contributing to increase food production and serving as a vital source of dietary protein for rural communities. In 2018, China contributed 479,164 metric tons to the global catch of *P. pelagicus*, accounting for nearly the entire global output of 493,134 metric tons that year (Duan *et al*. 2022; FAO 2022). However, in 2019, total landings from 17 countries declined to 313,420 metric tons (Lutz 2024). More recently, in 2022, Indonesia reported an annual catch of approximately 63 metric tons (Naviah *et al*. 2022).

Rising issues regarding to the low survival of crab larvae faced by aquafarmers nowadays lead to fewer viable juveniles for aquaculture, impacting overall production capacity (Alam *et al*. 2019). Larval survival is not only affected by susceptibility to diseases and infections, but also by non-synchronous moulting, which increases the risk of cannibalism and often leads to high mortality rates. Hence resulting in significant economic losses for crab hatcheries. If the current situation continues, crab farming will struggle to meet the demand for human consumption. Previous research has explored the use of synthetic hormones to accelerate moulting in crustacean larvae, and these artificial hormones have been used in aquaculture to promote crab growth. However, the use of artificial hormones has raised concerns, as they pose risks to aquatic organisms, the environment and human health through seafood consumption (Hajar-Azira *et al*. 2023). To reduce the use of artificial hormones, efforts are being made to find alternative plants that promote crab growth without relying on synthetic chemicals (Lu *et al*. 2023). The effectiveness of plants has been reported in many crustaceans, offering benefits like high efficacy, low toxicity, no hormone resistance and improved growth (Liao *et al*. 2023). For example, plant extracts such as soursop leaf, *Annona muricata*, guava leaf, *Psidium guajava*, fiddlehead fern, *Diplazium esculentum* and *Ginkgo biloba* leaf have been proven to improve the growth of crustaceans such as the mud crab, *Scylla paramamosain* (Yue *et al*. 2023), mud crab, *S. olivacea* (Ali *et al*. 2023), giant freshwater prawn, *Macrobrachium rosenbergii* (Hajar-Azira *et al*. 2023), and Pacific white shrimp, *Penaeus vannamei* (Liao *et al*. 2023), respectively. To address ongoing issues surrounding the availability of alternative hormonal growth stimulants, efforts have been made to identify cheap and eco-friendly plant-based alternatives. Therefore, spinach (*S. oleracea*) was utilised in this study to assess its impact on crab larval growth.

Spinach bioactive constituents offer multiple functions, including consisting of growth-promoting compounds that shorten the intermoult period and achieving optimal survival (Baniesmaeili *et al*. 2023). According to Huda-Faujan *et al*. (2023), spinach contains various bioactive constituents such as phenolics, saponins, coumarins, terpenoids, alkaloids and most importantly, steroids, which may contribute to growth in crustaceans. Plant steroids act as stimulants that induce molting in crustaceans, as demonstrated in several previous studies that used steroid-rich plants such as fiddlehead fern (*D. esculentum*) and black plum (*Vitex glabrata*) to induce molting in the giant freshwater prawn (*Macrobrachium rosenbergii*) and blue swimming crab (*P. pelagicus*), respectively (Hajar-Azira *et al*. 2023; Sorach *et al*. 2013). Thus, spinach have emerged as promising alternatives due to their bioactive compounds, however, the potential effects of spinach on the growth performance of crab larvae remain insufficiently studied. By enriching live feed (*Artemia*) of crab larvae with spinach will provide more nutritious food for larvae, helping to improve their survival and growth. As such, the objective of this study was to investigate the effects of spinach on the survival, specific growth rate, intermolt period and steroid hormone levels of larval *P. pelagicus*. The study is expected to reveal steroidal compounds in spinach, and spinach enrichment in live feed may enhance the survival, growth and steroid levels in *P. pelagicus* larvae.

## MATERIALS AND METHODS

### Spinach Sampling, Extraction and GC-MS Analysis

**FIGURE 1:**
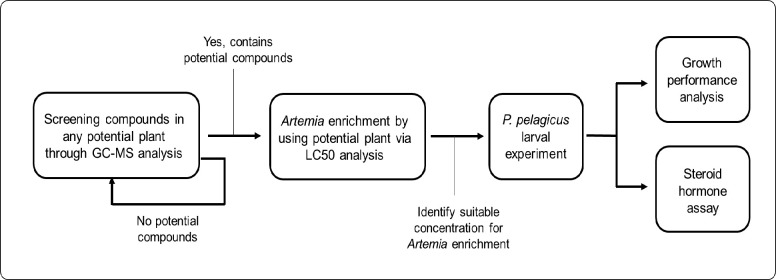
Overall experimental design was conducted throughout this study.

The overall experimental design was shown in Fig. 1 to further clarify the methodology used in this study. Spinach leaves used in this study were bought from Pasar Payang, Kuala Terengganu, Malaysia. The leaves were cut from the twigs using scissors and then transferred to the laboratory of Institute of Tropical Aquaculture and Fisheries (AKUATROP), Universiti Malaysia Terengganu (UMT), Terengganu, Malaysia. Fresh spinach leaves were weighed and washed using tap water, and the leaves were dried under shade in the open air, away from direct sunlight. The dried leaves were grounded and stored in a sterile, airtight container in the dark, at room temperature, for further use during *Artemia* LC_50_ analysis and larval experiment.

The spinach extraction was carried out at the laboratory of AKUATROP, UMT while GC-MS analysis was conducted at laboratory of Universiti Malaysia Pahang (UMP). Spinach leaves were extracted with 100 mL 80% methanol for 24 h by shaking with an orbital shaker (200 rpm and 27°C). The supernatant was then filtered through a filter paper, Whatman No.1. The filtrates were collected and evaporated using a rotary evaporator at 38°C under reduced pressure in a Buchi Rotavapor-R rotary evaporator. The crude extract was freeze-dried to obtain a dry extract to be used for *Artemia* enrichment. To screen for bioactive compounds, a portion of the crude extract powder was sent to UMP as an outsourced service for GC-MS analysis (Hewlett Packard GC-MS) using a Capillary column (model: Agilent 19091s-433 HP-5MS, pressure: 13.26 psi, flow: 1.2 mL/min, velocity: 29 cm/sec, temperature: 325°C) and helium as carrier gas. Triplicate measurements were taken for the crude extract powder. Quantification was not performed using external standards; thus, compound levels are expressed as relative peak area percentages, representing semi-quantitative estimates of compound abundance.

### Acute Toxicity Test LC50 (*Artemia* Enrichment)

Acute toxicity tests (48 h, LC_50_) of spinach powder were conducted on two-day-old *Artemia* (Great Salt Lake brand) according to Ayotunde *et al*. (2010), with slight modifications. Six different treatments (1,000 ppm, 1,200 ppm, 1,400 ppm, 1,600 ppm, 1,800 ppm and 2,000 ppm) of spinach powder were used in this experiment, with six replicates per treatment. The salinity, photoperiod, and temperature of the water were maintained at 30 ppt, 12L:12D and 28 ± 2°C, respectively. Salinity was determined using a hand refractometer (HTC, calibrated with distilled water). Neither feeding nor water changes were conducted throughout this experiment. At the end of this experiment, survival was recorded. The survival formula was calculated as follows.


Survival rate (%)=(Final amount of Artemia)/(Initial amount of Artemia)×100

Then, LC_50_ was revealed through mortality percentage, probit analysis and regression. The results obtained for *Artemia* LC_50_ was used to decide concentration of spinach-enriched *Artemia* for later larval experiment.

### Crab Sampling and Rearing

Twenty female matured *P. pelagicus* were obtained from the local fisherman that was collected at Pantai Remis, Perak and transferred into four-ton fibreglass tanks (holding tank) equipped with RAS system in the marine hatchery of AKUATROP, UMT, Terengganu, Malaysia. A 1-foot-thick layer of fine sand was spread on part of the holding tank bottom to promote spawning. The crabs were fed with chopped fish and squid at 20% of their wet body weight once per day at 1700 h–1900 h. Throughout the rearing of female *P. pelagicus*, the salinity, temperature, pH and dissolved oxygen of the water were maintained at 30 ppt–31 ppt, 28 ± 2°C, pH 7–7.9, and 5 ppm, respectively. Seawater was exchanged 50% every two weeks and every day for the holding tank and berried tank, respectively. Any excess food on the bottom tank was removed daily by a fishing net to prevent seawater contamination. The berried crab was checked daily, once the berried crab was noticed, the berried crab underwent prophylactic treatment by soaking with 150 ppm formalin solution for 30 min and then transferred individually into the berried tank (100 L). Once the berried females hatch (usually on days 12–15, depending on ambient temperature, 28°C–29°C) (Hidir *et al*. 2021), newly hatched larvae were collected and transferred into a 1,200 L fibreglass tank with a stocking density of 50 zoeas per litre for further experiments.

### Growth Performance

The *P. pelagicus* larvae were divided into two groups, one fed with non-enriched *Artemia* (0 ppm, control), and the other with spinach-enriched *Artemia* (concentration based on the LC_50_ experiment). The larvae were placed with 50 ind/L in each fibreglass tank (1,200 L) with five replicates per treatment. Larvae were fed thrice daily with *Artemia* (morning, afternoon and late evening) with an average rate of 2 *Artemia* larva^−1^ during Zoea 1 (Z1), while from Z2 to Z4, the feeding rate increased from 2 *Artemia* larva^−1^ to 5 *Artemia* larva^−1^. At the end of the larval stage (megalopa stage), the larvae were fed live *Artemia* with a feeding rate 5 *Artemia* larva^−1^.

Throughout the larval rearing experiment, salinity, temperature, pH and dissolved oxygen of the water were maintained at 30 ppt–31 ppt, 30°C–31°C, pH 7–7.9 and 5 ppm, respectively. The water parameter was examined using a YSI probe multiparameter. Meanwhile, the temperature was controlled by submersible thermostat-controlled heaters (500-watt). Seawater was exchanged 10% daily, at the same time, any excess food on the bottom tank was siphoned daily to prevent seawater contamination. The experiment was terminated when all megalopae had moulted to the crablet stage. Throughout this experiment, data on growth performance analyses was collected according to larval stages such as survival, specific growth rate (SGR), and intermolt period. To obtain the larval weight for SGR analysis, microbalance (Mettler Teledo MX5-0.000 mg) was used. All values of growth performance analyses were presented as mean ± standard deviation. The formula was calculated as follows (Ikhwanuddin *et al*. 2012; Hidir *et al*. 2021):

1. Survival rate (%) = (Final amount of larva)/(Initial amount of larva) × 100
2. Specific Growth Rate (% day^−1^) = ((In Final body weight – In Initial body weight)/culture period) × 100
3. Intermoult period (days) = Period time between two moult events

### Steroid Hormone

Larval samples from the Treatment 1,600 ppm and the control group were used for steroid analysis. A pooled sample of larvae (one-gram wet weight) was collected and weighed using Mettler Teledo MX5 microbalance (0.000 mg). For the extraction process, mixtures of 20 mL chloroform and deionised water were used during homogenising the samples, supernatant was collected after centrifuging at 5,000 rpm for 10 min, eluted with 25% methanol, and stored in the refrigerator (−20°C).

For steroid hormonal assay, steroids-extract supernatant was analysed using ELISA kit (Neogen Corporation, Canada) following the manufacturer’s protocol. The supernatant samples were then diluted with ELISA buffer in several different dilutions for the preliminary test to obtain a suitable sample dilution that was within the detection limit range of the hormone assays. Each treatment was assayed in three replicates. A microplate reader (Multiskan FC, Version 1.00.94) was used to read the plate at a wavelength of 450 nm. The absorbance reading of samples was altered to steroid concentration regarding the standard curve of known steroid concentration provided in the assay kit. Data of steroid levels were expressed as mean ± SD.

### Statistical Analysis

All statistical analyses were conducted using the Statistical Package for the Social Sciences (SPSS version 26). For *Artemia* survival, one-way analysis of variance (ANOVA) and Tukey›s multiple comparison tests were used to determine any differences between five treatments (1,000 ppm, 1,200 ppm, 1,400 ppm, 1,600 ppm, 1,800 ppm and 2,000 ppm). For the growth performance of *P. pelagicus* larvae, an unpaired t-test was used to analyse survival, SGR and intermolt period to determine any differences between 0 ppm (Control) and Treatment 1,500 ppm groups. Differences at *p* < 0.05 were considered significant.

## RESULTS

### Gas Chromatograph-Mass Analysis (GC-MS) for Spinach Leaf Extracts (*S. oleracea*)

Table 1 indicates the six highest percentage of compounds being screened through GC-MS. These six compounds are phenanthro[3,2-b]furan-7,11-dione, 1,2,3,4,8,9-hexahydro-4,4,8-trimethyl-, (+)-, n-Hexadecanoic acid, flavone, 5-hydroxy-4′-methoxy-7-methyl-, cyclononasiloxane, octadecamethyl-, octadec-9-enoic acid, stigmasta-5,22-dien-3-ol, acetate, (3.beta.,22Z)- (with areas of 6.97, 5.23, 3.61, 3.39, 2.53 and 2.15, respectively). GC-MS analyses determined four steroidal compounds such as stigmasta-5,22-dien-3-ol, acetate, (3.beta.,22Z)-, stigmastan-3,5-diene, a-Homocholest-4a-en-3-one and cholesta-3,5-dien-7-one and three fatty acid compounds such as n-Hexadecanoic acid, octadec-9-enoic acid and docosanoic acid.

### LC_50_ (*Artemia* Enrichment)

**FIGURE 2:**
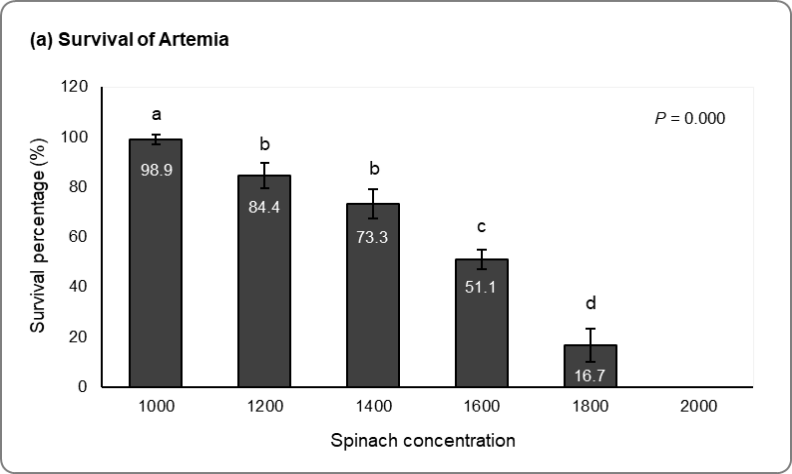
LC_50_ analysis of *Artemia* and *P. pelagicus*. (a) Survival percentage of *Artemia* enriched with varying spinach concentration. (b) Survival percentage of Zoea 1 fed with *Artemia* enrich with varying spinach concentration. ^a,b,c^ = Means with different superscripts are significantly different (ANOVA, Tukey test, *p* < 0.05), and the comparisons are made between different spinach concentration.

Fig. 2 illustrated spinach enrichment on *Artemia* resulted in lower survival with elevation spinach concentration from 1,000 ppm to 2,000 ppm (ANOVA, *p* = 0.000). 100% mortality of *Artemia* after exposure with 2,000 ppm for 24 h. LC_50_ calculation through probit analysis revealed LC_50_ was 1,621.81 ppm. Thus, the LC_50_ = 1,600 ppm was used as the treatment concentration in the later larval experiment.

### Growth Performance

**FIGURE 3:**
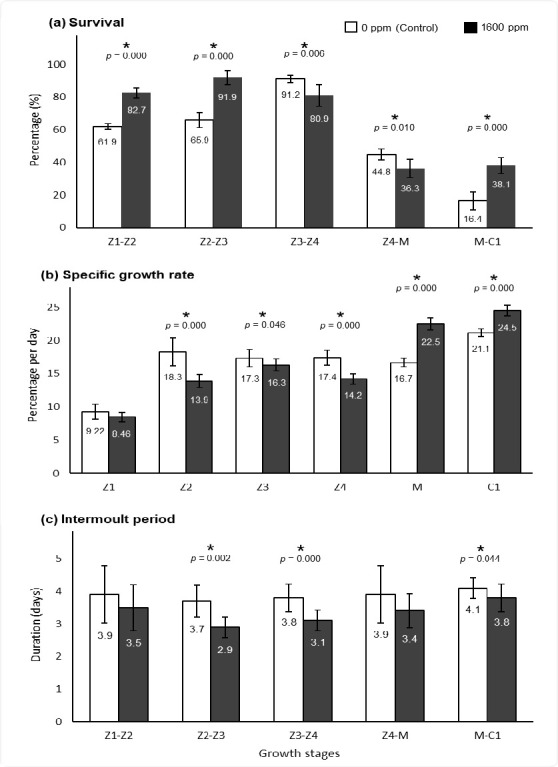
Spinach effect on growth performance of *P. pelagicus*. (a) Survival percentage, (b) specific growth rate, and (c) intermoult period. *Asterisk indicates significantly different (*t*-test, *p* < 0.05), and the comparisons are made between Control (no spinach) and Treatment 1,600 ppm.

**FIGURE 4:**
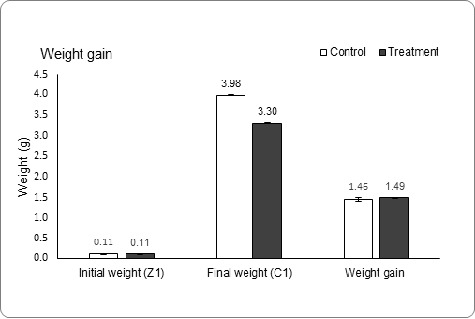
Spinach effect on growth performance of *P. pelagicus* including initial weight, final weight and weight gain. *Asterisk indicates significantly different (*t-*test, *p* < 0.05), and the comparisons are made between Control (no spinach) and Treatment 1,600 ppm.

Fig. 3a illustrated significantly higher survival for *P. pelagicus* in 1,600 ppm treatment for crab growth stages such as Z1–Z2 (82.6%), Z2–Z3 (91.9%) and M–C1(38.1%) compared to Control (Z1–Z2: 61.9%, Z2–Z3: 65.9% and M–C1: 16.3%) with *p* = 0.000 (*t*-test) for all analyses. Fig. 3b demonstrated higher growth stages of crab, indicating increment of SGR which Treatment 1,600 ppm higher than Control at the end growth stages such as Megalopa, M and Crablet 1, C1. The 1,600 ppm group reported M (22.5%) and C1 (24.5%) had significantly higher SGR compared to Control (M: 16.6%, C1: 21.1%) with *p* = 0.000, respectively (*t*-test). Fig. 3c displayed a shorter intermolt period in all growth stages for Treatment 1600 ppm than Control, primarily for Z2–Z3, Z3–Z4 and M–C1, as during these period stages reported significant differences between both Treatment 1,600 ppm and Control (*t*-test, *p* = 0.002, 0.000 and 0.044, respectively). In the Treatment 1,600 ppm, the intermoult period for Z2–Z3 (2.9 days), Z3–Z4 (3.1 days), and M–C1 (3.8 days) are shorter compared to Control with Z2–Z3, Z3–Z4 and M–C1 exhibited 3.7 days, 3.8 days and 4.1 days, respectively. Fig. 4 illustrated the initial weight, final weight and weight gain of *P. pelagicus*. There was no significant of weight gain between Treatment 1,600 ppm and Control.

### Steroid Hormone

**FIGURE 5:**
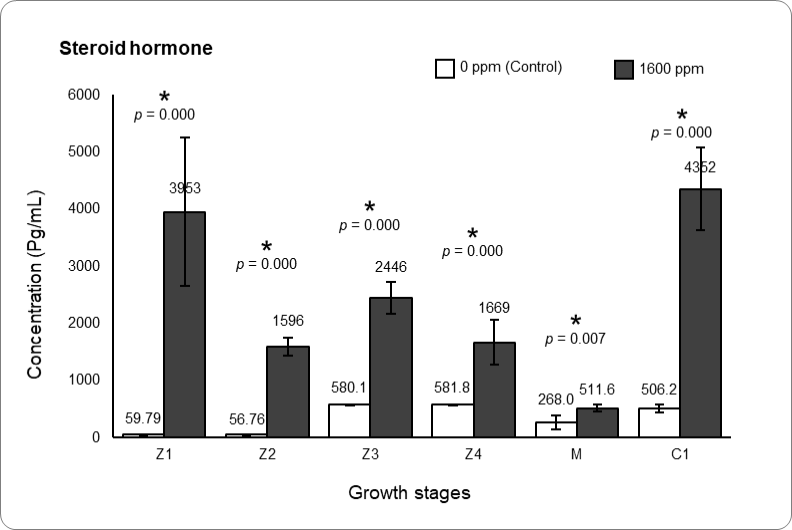
Spinach effect on steroid concentration in different *P. pelagicus* growth stages. *Asterisk indicates significantly different (*t*-test, *p* < 0.05), and the comparisons are made between Control (no spinach) and Treatment 1,600 ppm.

Fig. 5 illustrated steroid hormone levels for both Treatment 1,600 ppm and Control for all growth stages in *P. pelagicus* were significantly different with *p* < 0.001 (*t*-test). Z1, Z2, Z3, Z4 and C1 (511.63 pg/mL–3,953 pg/mL) in 1,600 ppm group had higher steroid hormone level compared to the Control group with range of steroid hormone levels ranging from 56.76 pg/mL to 581.80 pg/mL.

## DISCUSSION

In crab farming, the purpose of *Artemia* enrichment is to provide highly nutritious food for crab larvae, which helps improve their survival and growth. Without enrichment, relying solely on *Artemia* can hinder larval survival due to nutritional deficiencies. To enhance current enrichment methods, spinach may be considered as a cost-effective alternative to reduce dependence on expensive materials such as fish oil and phytoplankton. Therefore, this study began with an LC_50_ analysis on *Artemia* to assess their tolerance to varying spinach concentrations ranging from 1,000 to 2,000 ppm, and results indicated a steady decline in *Artemia* survival with increasing spinach concentration. The study stated that 1,000 ppm treatment had the highest survival, no survival was observed for 2,000 ppm treatments, and probit analysis demonstrated LC_50_ was at spinach concentration of 1621.81 ppm.

The LC_50_ result was used as a reference to select 1,600 ppm spinach concentration for enriching *Artemia*, which were fed to *P. pelagicus* larvae from the Z1 stage to the crablet stage. The purpose of using LC_50_ is to deliver the highest possible amount of bioactive compounds to the crab larvae without compromising *Artemia* survival. Two treatments were used in this study such as 1,600 ppm and 0 ppm (Control), 1,600 ppm recorded significantly higher survival for larval stages from Z1 to C1. SGR results demonstrated 1,600 ppm treatment higher than Control particularly, at the end growth stages encompassing M and C1 stages. Both results from survival and SGR were consistent with an intermoult period, where 1,600 ppm group had a shorter intermoult period for Z2 until C1 stages compared to Control. Overall, crab larvae fed with spinach-enriched *Artemia* exhibited improved growth performance in terms of survival, growth and molting than those in the control treatment. The expected result is likely attributed to bioactive compounds in spinach that support the growth of *P. pelagicus* larvae.

Through GC-MS analysis, spinach offers numerous potential compounds, such as steroids, that improve larval crab growth. The steroids being identified in the spinach such as stigmasta-5,22-dien-3-ol, acetate, (3.beta.,22Z), stigmastan-3,5-diene, a-Homocholest-4a-en-3-one and cholesta-3,5-dien-7-one. These steroids were classified as phytosterols, which carry out steroid function more efficiently in promoting growth in crabs (Iromo *et al*. 2021). To evaluate the efficacy of the steroidal compounds present in spinach, steroid assay results on crab larvae showed a consistent trend, with the 1,600 ppm group exhibiting higher steroid hormone levels in all larval stages than Controls. Previous research study by Baniesmaeili *et al*. (2023) stated that phytosterols in moringa leaf, *Moringa oleifera* such as Stigmast-7-en-3-ol and 4,22-Cholestadien-3-one improved the survival rate of Pacific white shrimp, *P. vannamei* (> 90%) than Control treatments (80.8%). Other research study also reported that the steroidal compounds in the Fiddlehead fern, *D. esculatum* contributed to higher survival (77.2%) and SGR (1.66% day^-1^) in the larvae of giant freshwater prawn, *Macrobrachium rosenbergii* after fed with dosages 10 g/kg compared to Control treatments (survival: 61.1%, SGR: 0.35% day^−1^) (Hajar-Azira *et al*. 2023). Also, the same study reported the post-larvae of giant freshwater prawns fed Fiddlehead fern showed a shorter moult cycle of about 29.9 days than the Control groups (31.9 days). Fujaya *et al*. (2019) reported that steroid-rich mulberry leaf extract, used at doses of 1.1 mg/g, 1.9 mg/g and 2.7 mg/g, increased the concentration of steroid compounds in the haemolymph of the mud crab (*Scylla olivacea*) to 1,065 ng/mL, 1,280 ng/mL and 1,592 ng/mL,, respectively. This increase corresponded with higher molting percentages of 50.0%, 56.6%, and 60.0%, respectively (Fujaya *et al*. 2019).

Steroidal compounds are not the sole contributors to the improved growth performance of larval crabs, high levels of fatty acids, such as hexadecanoic acid (palmitic acid) octadec-9-enoic acid (oleic acid), and docosanoic acid (behenic acid) present in spinach, may also play a significant role in promoting larval growth. Palmitic acid and behenic acid belong to the saturated fatty acid group, while oleic acid is classified as a monounsaturated fatty acid. According to Yu *et al*. (2020), palmitic acid is commonly found in plants and plays a crucial role in promoting cell growth and autophagy. It is also an essential component of phospholipids and serves as a precursor for other fatty acids (Yue *et al*. 2023). These functions may contribute to the enhanced growth performance of *P. pelagicus* larvae observed in the present study. Currently, there is limited information on whether these fatty acids influence growth performance in crustaceans but, a previous study has investigated the effects of oleic acid and behenic acid in fish. For example, Natnan *et al*. (2022) reported that grouper (*Epinephelus* sp.) fed diets supplemented with oleic acid (weight gain: 34.1 g; survival: 63.3%) and behenic acid (weight gain: 31.1 g; survival: 50.0%) exhibited higher weight gain and survival compared to the control group (weight gain: 26.0 g; survival: 43.3%). Taken together, both the present and previous studies highlight the importance of fatty acids, specifically palmitic acid, oleic acid and behenic acid in plant extracts, which appear to play a key role in growth of crab larvae.

Additionally, other compound determined in the spinach is a flavonoid, such as Flavone, 5-hydroxy-4′-methoxy-7-methyl-. Saptiani *et al*. (2021) and El-Refiae *et al*. (2024) examined flavonoid in mangrove leaf extract, *Acanthus ilicifolius* and Paulownia plants, *Paulownia* sp., respectively, which this compound played a vital antimicrobial role and promoted antioxidant activity which improved immunity in black tiger prawn, *Penaeus monodon* and Nile tilapia, *Oreochromis niloticus*, respectively. While this study did not directly assess the immune responses of *P. pelagicus* larvae, the known immunostimulatory effects of flavonoids suggest that such mechanisms may have contributed to the enhanced survival and growth observed. Although the manipulation in this experiment used spinach-enriched *Artemia*, other factors may also act as variables, such as culture conditions involving temperature, light, and water quality, which were kept as constant as possible. These environmental factors may influence the nutritional composition and overall health of crab larvae, potentially affecting the outcome and reliability of the experimental results.

## CONCLUSION

In conclusion, spinach contains essential bioactive compounds such as steroids, palmitic acid, oleic acid and flavonoid derivatives that contribute to the survival and growth performance of *P. pelagicus* larvae. Incorporating spinach as a natural supplement aligns with sustainable aquaculture practices by reducing reliance on synthetic steroids. Moreover, spinach supplementation is cost-effective and has potential for commercialisation in powdered or extract forms, offering a viable alternative to expensive phytoplankton or commercial enrichment products. However, spinach may be deficient in DHA and EPA, both of which are essential for optimal growth of crab larvae. Expanding the application of spinach at 1,600 ppm represents a promising strategy for commercial hatchery production of *P. pelagicus* in the future. To further strengthen the findings of the present study, it is recommended to conduct immunity-related analyses, including assessments of antimicrobial and antioxidant responses, to more evaluate the impact of spinach on larval health. The combination of spinach with other ingredients high in DHA and EPA may yield enhanced outcomes.

## Figures and Tables

**TABLE 1:** Compounds identified in the spinach, *Spinacia oleracea* leaf extract through GC-MS analysis.

No	Retention time	Compounds	Peak areas (%)	Nature compound
1	27.3678	Phenanthro[3,2-b]furan-7,11-dione, 1,2,3,4,8,9-hexahydro-4,4,8-trimethyl-, (+)-	6.971	Not found
2	19.2742	n-Hexadecanoic acid	5.203	Fatty acid
3	25.8633	Flavone, 5-hydroxy-4′-methoxy-7-methyl-	3.61	Flavonoid
4	21.8532	Cyclononasiloxane, octadecamethyl-	3.386	Silicone related group
5	21.0302	Octadec-9-enoic acid	2.53	Fatty acid
6	29.4122	Stigmasta-5,22-dien-3-ol, acetate, (3.beta.,22Z)-	2.154	Steroids
7	29.7896	Stigmastan-3,5-diene	1.726	Steroids
8	26.5395	Dienestrol	1.381	Synthetic estrogen
9	24.5423	Docosanoic acid	1.363	Fatty acid
10	29.7267	a-Homocholest-4a-en-3-one	1.176	Steroids
11	23.6984	Benzoic acid, 2-[(trimethylsilyl)amino]-, trimethylsilyl ester	1.161	Benzoic acid derivatives
No	Retention time	Compounds	Peak areas (%)	Nature compound
12	27.6299	Tricyclo [5.2.1.0(1,5)]dec-8-ene-6-carboxylic acid, 4-oxo-3-O-tolyl-10-oxa-3-aza-, pentyl ester	1.131	Not found
13	25.3811	Benzene, 1-phenyl-4-(2-cyano-2-phenylethenyl)	1.049	Benzene derivaties
14	29.0872	Cholesta-3,5-dien-7-one	0.875	Steroids
15	27.6875	1-(4-Chloro-phenyl)-5-[4-(1,1,2,2-tetrafluoro-ethoxy)-benzylsulfanyl]-1H-tetrazole	0.71	Not found
16	27.5198	Methanol, (1-ethyl-2-benzimidazolyl) (3,4-methylenedioxyphenyl)-	0.61	Methanol derivatives
17	23.1952	Propionitrile, 3-[1-[4-[1-(2-cyanoethoxy) cyclohexyl] buta-1,3-diynyl]cyclohexyl]-	0.503	Not found
18	17.8274	Benzothiazole, 2-(5-chloromethyl-1,3,4-oxadiazol-2-yl)-6-methoxy-	0.351	Not found
19	23.0169	4-Allyl-5-furan-2-yl-2,4-dihydro-[1,2,4]triazole-3-thione	0.325	Not found
20	22.9698	2-[5-(2,2-Dimethyl-6-methylene-cyclohexyl)-3-methyl-pent-2-enyl]-1,4-dimethoxy-benzene	0.163	Not found
